# Study of the microRNA expression profile of foreskin derived mesenchymal stromal cells following inflammation priming

**DOI:** 10.1186/s12967-016-1106-3

**Published:** 2017-01-13

**Authors:** Hussein Fayyad-Kazan, Mohammad Fayyad-Kazan, Bassam Badran, Dominique Bron, Laurence Lagneaux, Mehdi Najar

**Affiliations:** 1 Laboratory of Cancer Biology and Molecular Immunology, Faculty of Sciences I, Lebanese University, Hadath, Lebanon; 2 Institut de Biologie et de Médecine Moléculaires, Université Libre de Bruxelles, 6041 Gosselies, Belgium; 3 Laboratory of Clinical Cell Therapy, Institut Jules Bordet, Université Libre de Bruxelles (ULB), Campus Erasme, Brussels, Belgium

**Keywords:** MSCs, Foreskin, Inflammation, miRNA

## Abstract

**Background:**

Due to their self-renewal capacity, multi-lineage potential, and immunomodulatory properties, mesenchymal stromal cells (MSCs) are an attractive tool for different therapeutic strategies. Foreskin (FSK), considered as a biological waste material, has already been shown to be a valuable source of MSCs. Besides their typical fibroblast like morphology and International Society for cellular Therapy compliant phenotype, foreskin-MSCs (FSK–MSCs) are clonogenic, and highly proliferative cells with multi-lineage and strong immunomodulatory capacities. Of importance, FSK–MSCs properly adjust their fate following exposure to inflammatory signals. Being potent regulators of gene expression, miRNAs are involved in modulating nearly all cellular processes and in orchestrating the roles of different immune cells. In this study, we characterized the miRNome of FSK–MSCs by determining the expression profile of 380 different miRNAs in inflammation primed vs. control non-primed cells.

**Methods:**

TaqMan low density array (TLDA) was performed to identify dysregulated miRNAs after exposing FSK–MSCs to inflammatory signals. Quantitative real-time RT-PCR was carried out to validate the observations. DIANA-miRPath analysis web server was used to identify potential pathways that could be targeted by the dysregulated miRNAs.

**Results:**

Sixteen miRNAs were differentially expressed in inflammation-primed vs. non-primed FSK–MSCs. The expression level of miR-27a, -145, -149, -194, -199a, -221, -328, -345, -423-5p, -485-3p, -485-5p, -615-5p and -758 was downregulated whilst that of miR-155, -363 and -886-3p was upregulated. Target pathway prediction of those differentially expressed miRNAs identified different inflammation linked pathways.

**Conclusions:**

After determining their miRNome, we identified a striking effect of inflammatory signals on the miRNAs’ expression levels in FSK–MSCs. Our results highlight a potential role of miRNAs in modulating the transcription programs of FSK–MSCs in response to inflammatory signals. Further, we propose that specific miRNAs could serve as interesting targets to manipulate some functions of FSK–MSCs, thus ameliorating their therapeutic potential.

## Background

Mesenchymal stromal cells (MSCs) are multipotent fibroblast-like cells found in almost all tissues [1]. This multi-lineage potential, along with their capacity of supporting hematopoiesis and modulating immune responses are the main properties of MSCs. The simple and easy isolation procedures together with the great expansion potential, render MSCs as ideal candidates for different cellular therapies [2]. Despite some common characteristics and properties shared by MSCs of different origin [3], significant differences in their immunobiological features exist [4, 5]. Although it has long been considered as a biological waste material, foreskin (FSK) is, nowadays, considered as an important reservoir of therapeutic cells having potential value for several clinical applications [6, 7]. Recently, we have demonstrated that the foreskin is a new source of MSCs, designated as FSK–MSCs [8]. Being compliant with ISCT criteria, non-immunogenic and showing powerful immunomodulatory capacities [8], FSK–MSCs are highlighted as a valuable tolerogenic tool for cell-based immunotherapy. As known, MSCs are particularly sensitive to the local inflammatory microenvironment that modulate their functions and responses. Indeed, inflammation-priming leads to significant changes in the immunobiology of FSK–MSCs [8].

MicroRNAs (miRNAs) are small (19-22-nt) single-stranded noncoding RNA molecules that regulate the transcription or translation of the target gene mRNAs. Thus, they play crucial roles in many different cellular processes including cell differentiation, proliferation and immune homeostasis [9–12]. Accumulating data show that abnormal miRNA expression is a common feature of various diseases [9–12]. Nowadays, many evidences demonstrate a role for miRNAs in regulating distinct aspects of the immune system including cell proliferation, differentiation, fate determination and function mediated by different cell types [13–15]. The understanding of the miRNome of FSK–MSCs under inflammatory settings will help to identify the pattern of miRs that are modulated and that could serve as targets to enhance MSCs therapeutic functions. ***In this study***, comparative analysis showed that the miRNA expression profile of FSK–MSCs is critically different in inflammation primed vs. control non-primed cells. Our results reveal specific miRNA expression differences following inflammation priming and identified 16 differentially expressed miRNAs. Those altered miRNAs might be involved in the molecular mechanisms regulating FSK–MSCs immunobiology during inflammatory conditions and could be further used as targets to manipulate FSK–MSCs functions.

## Methods

### Ethical guidelines

This study was conducted in accordance with the Declaration of Helsinki (1964) and approved by the local ethics committee of the “Institut Jules Bordet” (Belgium). All donors and/or their parents or legal guardian were voluntary for giving the foreskin (FSK) samples as obtained following a circumcision procedure from healthy subjects. They gave written informed consent before specimen collection for using the samples and for publishing any associated scientific data. However, all personal information are kept confidential.

### Isolation, culture and characterization of FSK–MSCs

Briefly, after circumcision, foreskin was aseptically collected into a sterile specimen container containing sterile phosphate-buffered saline (PBS) buffer supplemented with penicillin/streptomycin. The samples were processed as previously published [8]. After a sterile wash with PBS, the sample was transferred into a petri dish where epidermis was manually removed from the skin and the dermis was cut into small pieces. By using Liberase Research Grade solution (Roche Diagnostics, Belgium), the tissue was digested and the resulting cell suspension was washed by centrifugation (800*g*, 5 min) in Dulbecco’s modified Eagle’s medium with low glucose (DMEM-LG; Lonza, Belguim) and containing 10% fetal bovine serum (FBS; Sigma-Aldrich, Belgium). The subsequent cell pellet was seeded in culture flasks with DMEM-LG (Lonza) supplemented with 10% FBS (Sigma-Aldrich), 2 mM l-glutamine and 50 U/ml penicillin (both from Lonza) and the cultures were incubated at 37 °C in a 5% CO2 humidified atmosphere. Non-adherent cells were removed when the medium was changed (once a week). When sub-confluence (80–90%) was achieved, adherent cells were harvested by TrypLE Select (Lonza) and expanded until the desired passage.

The cells were characterized according to the ISCT criteria. Thus, the immunophenotype was analysed by flow cytometry (MacsQuant analyzer (Miltenyi Biotec, Netherlands)) using fluorochrome labelled monoclonal antibodies: anti-CD45-FITC and anti-HLA-Dr-PE (Exalpha Biologicals, Maynard, MA), anti-CD34-PE and anti-CD73-PE (BD Biosciences, San Diego, CA, USA), anti-CD14-PE, anti-CD19-PE, anti-CD105-FITC and anti-CD90-PE (R&D systems, Minneapolis, MN, USA). Their multilineage potential was confirmed by inducing specific lineage commitment (adipogenic, osteogenic and chondrogenic differentiation) using appropriate induction culture medium (NH media, Miltenyi Biotec). Specific lineage commitment was highlighted by using lineage-specific cell staining techniques and microscopic examination.

### Inflammation priming of FSK–MSCs

Foreskin–mesenchymal stromal cells were analyzed under both constitutive and inflammatory conditions. Inflammation priming of FSK–MSCs was performed as previously described [16]. Briefly, cells were treated (overnight) with a pro-inflammatory cytokine cocktail containing IL-1β (Peprotech, Rocky Hill, NJ, USA) (25 ng/ml), TNF-α (50 ng/ml), IFN-α (3000 U/ml or 10 ng/ml) and IFN-γ (1000 U/ml or 50 ng/ml) (all from Prospec Inc., Rehovot, Israel). After priming, the medium was removed, and the cells were washed and became available for analysis.

### MiRNA expression profile

Total RNA was extracted from cells using TRIzol total RNA isolation reagent (Roche Applied Science). The concentration was quantified using a NanoDrop spectrophotometer. A three-step procedure was performed to profile the miRNAs. First, for cDNA synthesis from the miRNAs, 30 ng of total RNA was subjected to RT (reverse transcription) using a TaqMan® microRNA Reverse Transcription Kit (#4366596; Applied Biosystems) and Megaplex RT primers (Human Pool A, #4399966; Applied Biosystems) following the manufacturer’s protocol, allowing simultaneous reverse transcription of 380 mature human miRNAs to generate a miRNA cDNA library corresponding to each plasma sample. RT was performed on a Mastercycler Epgradient thermocycler (Eppendorf) with the following cycling conditions: 40 cycles at 16 °C for 2 min, 42 °C for 1 min and 50 °C for 1 s followed by a final step of 80 °C for 5 min to inactivate reverse transcriptase. Thereafter, to generate enough miRNA cDNA template for the following real-time PCR, the cDNA libraries were pre-amplified using Megaplex PreAmp primer (Humam Pool A, #4399233; Applied Biosystems) and PreAmp Master Mix (#4384266; AppliedBiosystems) following the manufacturer’s instructions. The PreAmp primer pool used here consisted of forward primers specific for each of the 380 human miRNAs and a universal reverse primer. The pre-amplification cycling conditions were as follows: 95 °C for 10 min, 55 °C for 2 min, 72 °C for 2 min followed by 12 cycles at 95 °C for 30 s and 60 °C for 4 min; the samples were then held at 99.9 °C for 10 min. After the pre-amplification step, the products were diluted with RNase-free water, combined with TaqMan gene expression Master Mix and then loaded into TaqMan Human MicroRNA Array A (#4398965; Applied Biosystems), which is a 384-well formatted plate and real-time PCR-based microfluidic card with embedded TaqMan primers and probes in each well for the 380 different mature human miRNAs. Real-time PCR was performed on an ABI PRISM 7900HT sequence detection system (Applied Biosystems) with the following cycling conditions: 50 °C for 2 min, 94.5 °C for 10 min followed by 40 cycles at 95 °C for 30 s and 59.7 °C for 1 min. The Ct (cycle threshold) was automatically given by SDS 2.4 software (Applied Biosystems) and is defined as the fractional cycle number at which the fluorescence passes the fixed threshold of 0.2. RNU48 embedded in the TaqMan human microRNA arrays was used as an endogenous control. The relative expression levels of miRNAs were calculated using the comparative ΔΔCt method as described previously [17, 18]. The fold changes in miRNAs were calculated by the equation 2^−ΔΔCt^.

### Taqman miRNA assay for individual miRNAs

Gene-specific reverse transcription was performed for each miR using 10 ng of purified total RNA, 100 mM dNTPs, 50 U MultiScribe reverse transcriptase, 20 U RNase inhibitor, and 50 nM of gene-specific RT primer samples using the TaqMan MicroRNA Reverse Transcription kit (Applied Biosystems, Gent, Belgium). 15 μl reactions were incubated for 30 min at 16 °C, 30 min at 42 °C, and 5 min at 85 °C to inactivate the reverse transcriptase. Real time RT-PCR reactions (5 μl of RT product, 10 μl TaqMan 2 × Universal PCR master Mix, (Applied Biosystems, Gent, Belgium), and 1 μl TaqMan MicroRNA Assay Mix containing PCR primers and TaqMan probes) were carried out on ABI Prism 7900HT Sequence Detection System (Applied Biosystems, Gent, Belgium) at 95 °C for 10 min followed by 40 cycles at 95 °C for 15 s and 60 °C for 1 min. The qRT-PCR reactions were performed in triplicate, and the signal was collected at the end of every cycle. The Ct (cycle threshold) was automatically given by SDS 2.4 software (Applied Biosystems) and is defined as the fractional cycle number at which the fluorescence passes the fixed threshold of 0.2.

RNU48 was used as an endogenous control. The relative expression levels of miRNAs were calculated using the comparative ΔΔCt method as described previously [31, 32]. The fold changes in miRNAs were calculated by the equation 2^−ΔΔCt^.

### Pathway enrichment analysis

Pathway enrichment analysis was employed to investigate the regulatory mechanisms of significantly differentially expressed miRNAs. DIANA-miRPath v.3.0 analysis web server was used for pathway enrichment analyses. The enriched pathways were defined by their enrichment of significantly differentially expressed miRNA target genes.

### Statistical analysis

Data analyses were performed using the SDS RQ Manager 1.2 software and DataAssist v2.0 software (Applied Biosystems). Statistical significance of miRNA expression between control and treated cells was determined according to unpaired Mann–Whitney U test. p Values <0.05 (*), <0.01 (**) were considered significant.

## Results

### Obtaining and characterizing FSK–MSCs

In order to obtain FSK–MSCs, the foreskin sample was first processed to separate the epidermis from the dermis (see "Methods" section) and then scraping was applied to obtain small tissue pieces from the dermis. Following enzymatic digestion, the tissue mixture was centrifuged and the resulting cell pellet was incubated for culture. After removing non adherent cells, colonies of cells with fibroblastic morphology started to appear. When sub-confluency (80–90%) was reached, adherent cells were harvested using TrypLE Select solution and expanded by successive passages.

These adherent cells were then characterized according to the ISCT criteria. Flow cytometry analysis demonstrated that those cells are positive (>95%) for the MSC markers CD73, CD90 and CD105 whilst negative (<5%) for CD45, CD34, CD14, CD19 and HLA-DR markers. It is noteworthy that inflammation priming had no significant impact on the expression level of those markers (data not shown).

To comply with the ISCT criteria, we further confirmed that the obtained cells exhibited a multi-lineage potential since they were able to differentiate into cells of adipogenic, osteogenic or chondrogenic lineage after being cultured with appropriate induction media (data not shown). After 21 days of osteogenic differentiation, a mineralization matrix following calcium deposits was observed by Alizarin red staining demonstrating the generation of osteoblasts. After 10 days of adipogenic differentiation, cells with cytoplasm containing several lipid vacuoles were revealed by Oil Red O staining indicating the formation of adipocytes. After 21 days of chondrogenic differentiation, the production of proteoglycans-based extracellular matrix was shown by Alcian blue staining illustrating the generation of chondrocytes.

### Impact of inflammation priming on miRNA-expression profile in FSK–MSCs cells

**Fig. 1:**
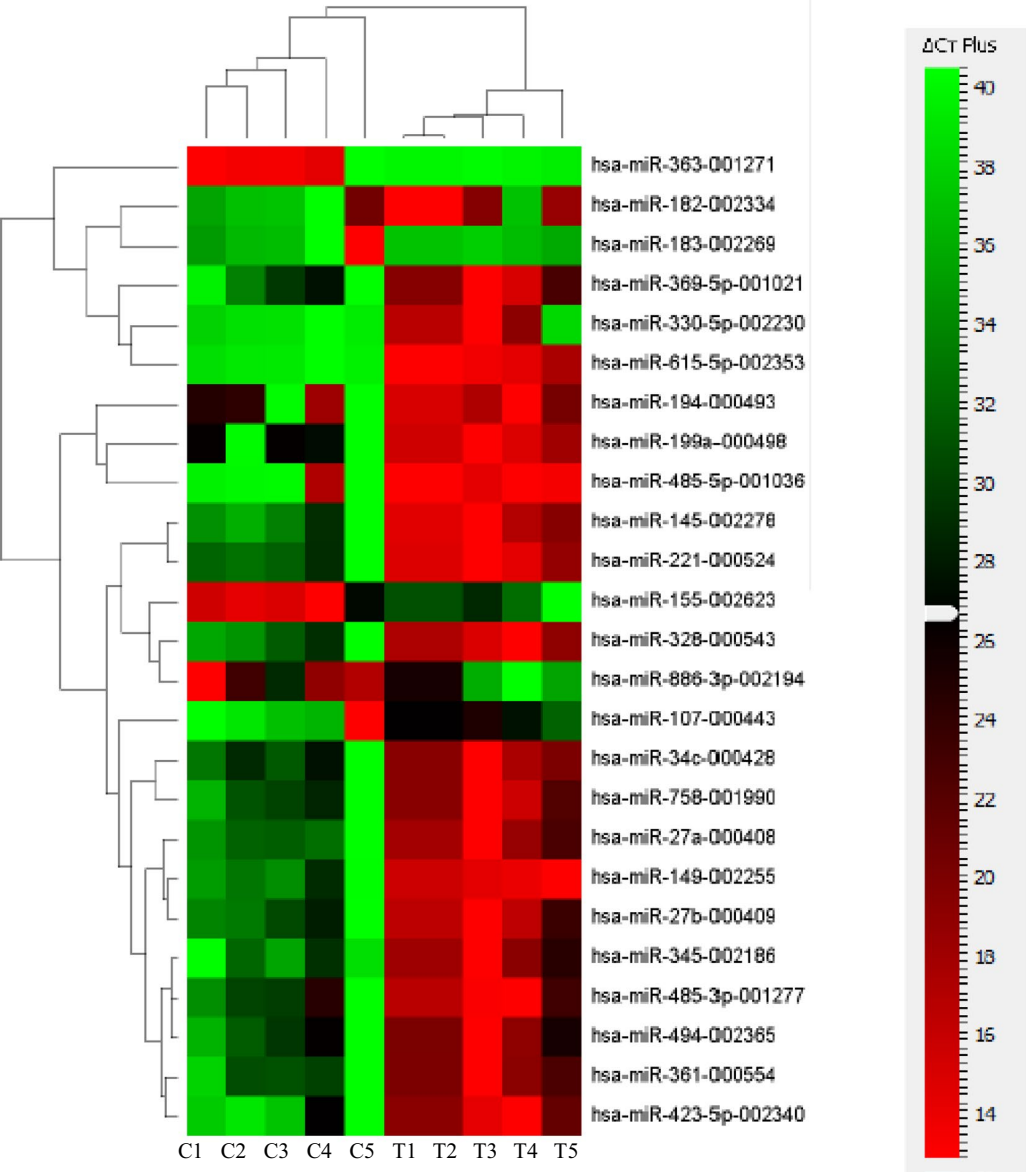
Differentially expressed miRNAs in inflammation-primed FSK–MSCs. Heat map presenting the differentially expressed miRNAs in inflammation-primed FSK–MSCs compared to their respective untreated partners (n = 5 in each group). MiRNA expression data was generated by performing TaqMan low-density arrays (TLDA). *Columns* correspond to inflammation-cocktail treated samples (T) or untreated control samples (C). Each *row* corresponds to an individual miRNA sequence. Only miRNAs significantly modulated (p < 0.05) are included in the map. The *colors* display miRNA expression variance where *red* indicates an increased abundance of miRNA in the indicated samples whereas *green* indicates a reduced miRNA level

Aware that MSCs can serve as sensors and switchers of inflammation, and given the importance of miRNAs in regulating inflammatory responses, we aimed at characterizing the expression level of different miRNAs in FSK–MSCs and investigating the impact of inflammation on this expression. FSK–MSCs were derived from 5 independent donors and total RNA was prepared from those cells being either untreated (control cells) or treated with a pro-inflammatory cytokine cocktail (treated cells). TaqMan low density array (TLDA) was then carried out to assess miRNA expression level in those cells. Before measuring miRNAs’ levels and in order to identify suitable endogenous controls, three different candidate miRNAs (RNU44, RN48, and U6 snRNA) were analyzed for variance in gene expression using Data Assist software v2.0. The statistical method ranked the candidate endogenous control genes with an excellent correlation of raw stability values (data not shown). The most stably expressed RNU48 was chosen as the endogenous control, and relative miRNA expression was normalized against RNU48. Our TLDA analysis revealed that the miRNA expression profile in treated cells is not totally similar to that of untreated cells. In fact, we show the expression level of 25 miRNAs that were modulated in treated vs. control cells (Fig. 1). Among these, 20 miRNAs (miR-145, -149, -182, -194, -199a, -221, -27a/b, -328, -330-5p, 345, -34c, -361, -369-5p, -423-5p, 485-3p, 485-5p, -494, -615-5p and-758) were downregulated whilst 5 miRNAs (miR-107, -155, -183, -363 and -886-3p) were upregulated. Details of the results are shown in Table 1. A clustergram of the samples as well as the significantly differentially expressed miRNAs in control and treated cells are illustrated in Fig. 1 in the form of a heat map generated using ΔCt values. Heat map is a typical method used in gene-expression analysis where the expression level of many genes across a number of tested samples can be displayed in a two dimensional image with a color code representing the expression intensity of each gene.

**Table 1:** MiRNA signature identified by TLDA Technique

microRNA	Inflammation vs. Ctrl ratio	p value
miR-145	0.022	0.012
miR-149	0.24	0.0044
miR-182	0.216	0.047
miR-194	0.221	0.039
miR-199a	0.032	0.031
miR-221	0.075	0.026
miR-27a	0.082	0.039
miR-27b	0.23	0.04
miR-328	0.385	0.023
miR-330-5p	0.0045	0.045
miR-345	0.12	0.046
miR-34c	0.0867	0.044
miR-361	0.1878	0.047
miR-369-5p	0.0213	0.041
miR-423-5p	0.296	0.0108
miR-485-3p	0.392	0.025
miR-485-5p	0.12	0.034
miR-494	0.27	0.046
miR-615-5p	0.004	0.042
miR-758	0.011	0.027
miR-107	12.5	0.048
miR-155	8.5	0.0081
miR-183	9.5	0.046
miR-363	15	0.013
miR-886-3p	3.5	0.02

Our TLDA analysis identified 25 miRNAs to be differentially expressed in treated vs. untreated control cells with a p value <0.05

The numbers corresponding to these colors are the ΔCt values. The dendrogram on the left side of the heat map classifies miRNAs into groups based on the divergence of miRNA expression values among the different samples. The dendrogram presented at the top indicates the relatedness of the samples based on overall miRNA expression values and separates the control from the treated group of samples.

**Fig. 2:**
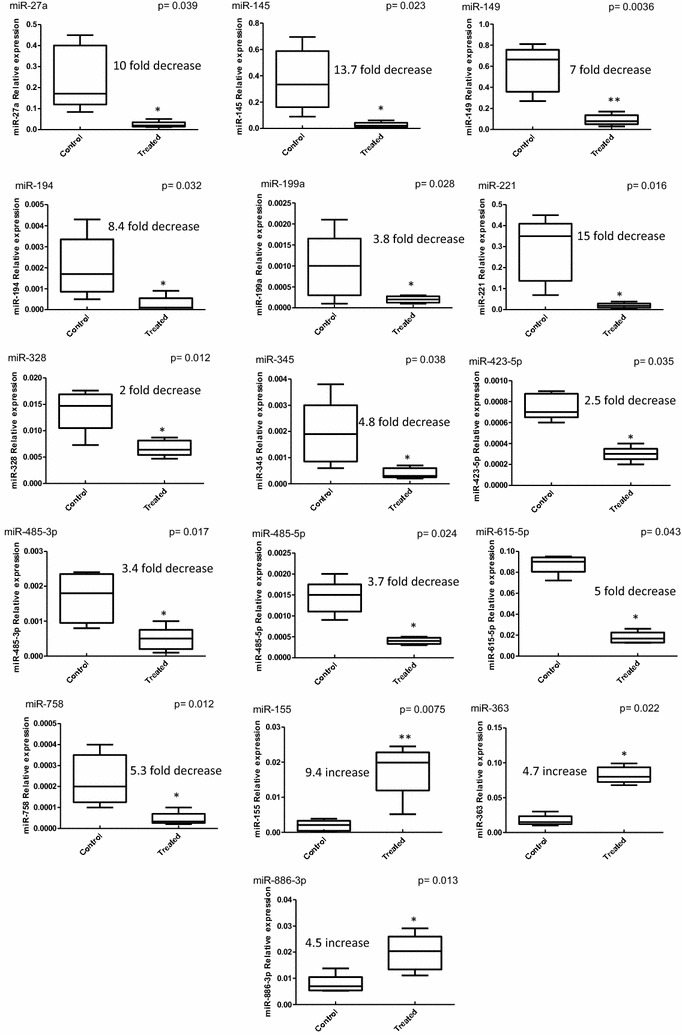
Sixteen miRNAs are differentially expressed after inflammation priming of FSK–MSCs. FSK–MSCs, derived from 5 independent donors, were cultivated in the absence or presence of inflammatory cocktail. *RNU48*-normalized miRNA levels were quantified by qRT-PCR and plotted as *Box plots*. The statistical significance was determined using Mann–Whitney U- test (*p <  0.05, **p < 0.01 vs. untreated control cells)

In a second step, and in order to validate their differential expression, miRNAs that appeared to be upregulated or downregulated in treated vs. control cells were further examined using individual quantitative Real Time PCR (qRT-PCR). Interestingly, out of the 25 miRNAs that showed altered expression (Table 1), 16 miRNAs were confirmed to exhibit such differential expression in treated vs. control cells (Fig. 2). Those 16 miRNAs fall in two groups. Group 1 contains 13 miRs that were downregulated (ratio between 0.1and 0.005) in treated cells in comparison to control cells and includes miR-27a, -145, -149, -194, -199a, -221, -328, -345, -423-5p, -485-3p, -485-5p, -615-5p and -758 (Fig. 2). Observing that those 16 miRs are not equal in terms of their downregulation rate led us to further classify them into subgroups. Group 1A corresponds to miRNAs that were most strikingly downregulated and consists of miR-27a, -145 and -221 that decreased 10, 13.7 and 15 folds, respectively. Group 1B consists of miRNAs that were less strikingly downregulated and includes miR-149, -194, -615-5p and -758 that exhibited decreased rates of 7, 8.4, 5 and 5.3 folds, respectively. Group 1C contains the least strongly downregulated miRNAs and includes miR-199a, -328, -345, -423-5p, 485-3p and -485-5p that showed downregulation rates of 3.8, 2, 4.8, 2.5, 3.4 and 3.7 folds, respectively.

On the other hand, group 2 contains 3 miRNAs (miR-155, -363 and -886-3p) that were upregulated (ratio greater than 3) in treated vs. control cells (Fig. 2). Among these, miR-155 was the most strikingly upregulated miR exhibiting a 9.4 fold increase whilst miR-363 and -886-3p showed increased rates of 4.7 and 4.5 folds, respectively. Altogether, these observations demonstrate a clear difference in the miRNA expression profile in FSK–MSCs exposed to inflammatory signals vs. control cells suggesting a potential role for miRNAs in modulating FSK–MSCs’ transcriptional programs in response to inflammatory conditions.

### Analysis of inflammation primed MSCs–associated miRNA pathways

Since each miRNA can regulate the expression of many target genes but multiple miRNAs can modulate specific pathways, we explored the pathways that were potentially regulated by the miRNAs observed to be altered in inflammation primed FSK–MSCs in comparison to untreated control MSCs.

For the identification of these pathways, DIANA-miRPath v.3.0 analysis web server was used and each of the statistically differentially expressed miRNAs was used as a query. The output Kyoto encyclopedia of genes and genomes (KEGG) pathways obtained for each miR included many results and for reasons of simplicity and specificity we focused only on pathways linked to inflammation. These pathways were previously summarized in [19] and included Adhesion-Extravasation-Migration, apoptosis signaling, calcium signaling, cytokine signaling, leukocyte signaling, innate pathogen detection, MAPK signaling, NF-κB signaling, PI3 K-AKT signaling, TNF superfamily signaling, NK cell signaling, GPCR signaling, ROS/Glutathione/cytotoxic granules, phagocytosis-antigen presentation, complement cascade, Eicosanoid signaling and Glucocorticoid signaling/PPAR signaling. The pathways targeted by upregulated and downregulated miRs are shown in Table 2. It is noteworthy that no KEGG pathways linked to inflammation were identified for miR-149, -328, -345, -363, -423-5p, -615-5p, -758 and -886-3p.

**Table 2:** Significantly enriched KEGG pathways (p < 0.05) targeted by miRNAs

miRNA	Term_ID	Term_name	Gene_count	p value
hsa-miR-27a-3p	hsa04520	Adherens junction	21	0.01
hsa-miR-27a-3p	hsa04151	PI3-AKT signaling pathway	78	0.03
hsa-miR-27a-3p	hsa-04115	Apoptosis signaling pathway	21	0.01
hsa-miR-145	hsa04310	Wnt/Ca^2+^ signaling pathway	40	0.02
hsa-miR-145	hsa04520	Adherens junction	32	6.9 x 10^−8^
hsa-miR-145	hsa04151	PI3-AKT signaling pathway	99	0.005
hsa-miR-145	hsa-04115	Apoptosis signaling pathway	26	0.005
hsa-miR-199a-5p	hsa04520	Adherens junction	8	0.001
hsa-miR-194-5p	hsa04520	Adherens junction	8	0.0004
hsa-miR-221-3p	hsa-04115	Apoptosis signaling pathway	13	0.007
hsa-miR-221-3p	hsa04670	Leukocyte transendothelial migration	14	0.02
hsa-miR-423-5p	hsa04520	Adherens junction	14	0.03
hsa-miR-485-5p	hsa04310	Wnt/Ca^2+^ signaling pathway	7	0.02
hsa-miR-155	hsa04520	Adherens junction	13	0.01
hsa-miR-155	hsa04064	NF-Kappa B signaling	16	0.002
hsa-miR-155	mmu04668	TNF signaling pathway	22	0.001
hsa-miR-155	hsa04520	Adherens junction	13	0.01

KEGG pathways, number of target genes, and p values were identified by DIANA miRPath v.3.0 for statistically significant differentially expressed miRNAs in inflammation primed FSK–MSCs vs. untreated control cells

## Discussion

In addition to their self-renewing and multi-lineage differentiation properties, MSCs exhibit immunoregulatory abilities through influencing both immune and inflammatory responses [20]. In fact, MSCs, can actively sense the surrounding microenvironment or inflammatory milieu and adopt their phenotype and responses, accordingly [20]. For instance, and depending on the environmental signals, MSCs can play both pro- and anti-inflammatory roles [20]. This marked functional plasticity of MSCs renders them as clinically relevant cell types with important therapeutic potential for tissue repair and regeneration as well as for the treatment of different inflammatory diseases and malignancies [20, 21]. Nowadays, different populations of MSCs, derived from distinct human tissues, are available and exhibit dissimilar immunological profiles and functional capacities [5, 22, 23]. FSK–MSCs are emerging as a highly interesting MSC-type due to their recently characterized properties [8]. Besides, FSK–MSCs, when exposed to inflammatory conditions, have been shown to exhibit induced expression of different immunoregulatory genes and factors [8]. Their high sensitivity to inflammation increased the interest in understanding the molecular mechanisms involved in coupling FSK–MSCs’ responses to the surrounding inflammatory microenvironment.

Among the different regulatory elements that controls gene expression and modulates cellular fate and behavior are miRNAs. For instance, they can significantly alter the responses of different innate and adaptive immune cells where any disturbance of their expression profiles can lead to several diseases [24–27]. Therefore, miRNAs are, nowadays, considered as attractive therapeutic targets [24–27]. Besides their ability to modulate immune responses, miRNAs play a key role in MSCs’ biology. For example, miRNAs can strikingly affect MSCs’ decision to either proliferate or differentiate into either cell lineage including adipogenic, chondrogenic, myogenic, neurogenic or other lineages [28]. The aim of this work was to determine the miRNome of FSK–MSCs by investigating the expression profile of 380 different miRNAs in inflammation primed vs. control non-primed cells. The major outcome of this study is our demonstration of a significantly altered miRNA expression profile in inflammation-primed FSK–MSCs where 16 miRNAs were found to be differentially expressed. This observed inflammation-triggered alteration of miRNA expression pattern is not surprising especially that different miRNAs have been reported to show altered expression in response to inflammatory signals. For example, miR-146 expression level is described to be upregulated in response to inflammatory stimuli [29], whilst other miRNAs including miR-9, -101, -125b, -155, -146, -192 and -203 are described to exhibit altered expression profiles in some inflammatory diseases [30].

We classified our differentially expressed miRNAs in two major groups: (1) a first one containing the miRNAs being downregulated in inflammation-primed FSK–MSCs and includes miR-27a, -145, -149, -194, -199a, -221, -328, -345, -423-5p, -485-3p, -485-5p, -615-5p and -758; (2) and a second one bearing the upregulated miRNAs and consists of miR-155, -363 and -886-3p. Interestingly, among those identified miRNAs, there are ones that have already been demonstrated to regulate MSCs’ behavior. For instance, miR-27a has been reported to regulate osteogenesis of MSCs [31] whilst miR-145, -194, and -199a have been described to regulate their chondrogenesis [32–34]. Moreover, miR-221 has been shown to be involved in the adipogenesis process of MSCs [35]. Besides its ability to regulate adipogenesis [35], miR-155 has also been described to modulate the responses of MSCs to inflammatory signals [36]. Furthermore, miR-886-3p overexpression has been shown to inhibit MSCs migration [37].

Given that a variety of genes, encoding cell adhesion molecules, co-stimulatory elements as well as immunoregulatory cytokines and factors, are modulated in inflammation-primed FSK–MSCs, it is strongly possible that the differentially expressed miRNAs could be involved in the regulatory processes accounting for such altered transcription programs in inflammation-primed vs. non-primed FSK–MSCs. This proposal is strongly supported by the observation that miR-155 expression can be modulated upon inflammation-priming of mouse MSCs and that miR-155 expression levels are tightly related to its ability to modulate the immunosuppressive capacity of those MSCs [36], thus providing a striking evidence for the importance of miRNAs in regulating the immunoregulatory capacity of MSCs. To get more insight into the potential mechanisms and signaling events that could be targeted by our differentially expressed miRNAs, we searched for the predicted target pathways of those miRNAs. In fact, given that multiple miRNAs may cooperate to regulate related target genes in a certain signaling route [38, 39], analysis of target pathways instead of individual genes was carried out. The miRNAs, being downregulated in inflammation-primed FSK–MSCs, are predicted to target pathways enriched in genes involved in Adherens junction, PI3-AKT pathway, apoptosis pathway, Wnt/Ca^2+^ pathway and leukocyte trans-endothelial migration. On the other hand, adherens junction, TNF and NF-KB signaling pathways are predicted to be the targets of miR-155 which is upregulated in inflammation-primed FSK–MSCs. Indeed, it was previously reported that miR-155, being upregulated in inflammation primed mouse MSCs, modulates MSCs immunosuppressive capacity upon modulating NF-KB activity [36].

The observed altered miRNA expression profile in inflammation-primed vs. non-primed FSK–MSCs imposes a detailed mechanistic characterization of the involvement of each of the identified miRNAs as well as their predicted pathway(s) on FSK–MSCs inflammatory responses in future studies. However, our observations might provide an early step toward developing a novel strategy to improve the immunosuppressive activity of FSK–MSCs. For instance, identifying the miRNAs that either dampen or trigger the expression of genes encoding immunosuppressive cytokines, factors and receptors would enable us to trigger, in vitro, the immunomodulatory capacity of FSK–MSCs by downregulating or upregulating the expression of certain miRNAs. This, in vitro, pre-activation of FSK–MSCs would greatly improve their therapeutic potential and medical value.

## Conclusions

In this report, we show that inflammation-priming of FSK–MSCs substantially alters their miRNA expression profile where 13 miRNAs are downregulated and 3 others are upregulated. Those differentially expressed miRNAs are predicted to target several candidate signaling pathways being involved in regulating cellular behavior in response to inflammatory signals.

Aware that the in vivo communication between grafted MSCs and the host inflammatory microenvironment orchestrates the plasticity of the immunological properties of the grafted MSCs, understanding the molecular events that transplanted MSCs undergo upon exposure to an inflammatory signal is of great importance. In this report, we highlight a potential role for miRNAs in regulating FSK–MSCs’ responses to inflammatory signals. Further, we suggest that miRNAs could serve as potential targets to improve, in vitro, the therapeutic value of MSCs before being grafted into the patient.

## Abbreviations

FSK: foreskin
GPCR: G protein-coupled receptor
IL: interleukin
IFN: interferon
ISCT: International Society for cellular Therapy
KEGG: Kyoto encyclopedia of genes and genomes
miR: microRNA
MSCs: mesenchymal stromal cells
PBS: phosphate-buffered saline
PPAR: peroxisome proliferator-activated receptor
*qRT*-*PCR*: *quantitative Real*-*Time PCR*
TNF: tumor necrosis factor
TLDA: TaqMan low density array
